# Anxiety and Depression Symptoms and Suicidal Ideation in Japan Rugby Top League Players

**DOI:** 10.3390/ijerph18031205

**Published:** 2021-01-29

**Authors:** Yasutaka Ojio, Asami Matsunaga, Kensuke Hatakeyama, Shin Kawamura, Masanori Horiguchi, Goro Yoshitani, Ayako Kanie, Masaru Horikoshi, Chiyo Fujii

**Affiliations:** 1 Department of Community Mental Health & Law, National Institute of Mental Health, National Center of Neurology and Psychiatry, Tokyo 187-8553, Japan; asamim@ncnp.go.jp (A.M.); chyfujii@ncnp.go.jp (C.F.); 2 Japan Rugby Players’ Association, Tokyo 108-0074, Japan; hatake.no.3@gmail.com (K.H.); shinkawamurashin@gmail.com (S.K.); hori21@bc.iij4u.or.jp (M.H.); yoshiath4@gmail.com (G.Y.); 3 National Center for Cognitive Behavioral Therapy and Research, National Center of Neurology and Psychiatry, Tokyo 187-8551, Japan; ayakokanie85@yahoo.co.jp (A.K.); mhorikoshi@ncnp.go.jp (M.H.)

**Keywords:** mental health, athletes, rugby, depression, anxiety, suicidal ideation

## Abstract

Clinical and research interest is growing in mental health support for elite athletes, based on findings from epidemiological surveys conducted in Australia, the United States, and European countries. However, little is known about the mental health status of elite athletes in Asia, including Japan. In the current study, we examine the prevalence of mental health problems and suicidal ideation and its risk factors in Japan Rugby Top League players. We analyze anonymous web-based self-reported data from 251 currently competing Japan Rugby Top League male players. During the off-season from December 2019 to January 2020, data on anxiety and depression symptoms were collected using the Japanese version of the 6-item Kessler-6. Suicidal ideation was assessed using the Baron Depression Screener for Athletes. Among the players, 81 players (32.3%) had experienced symptoms of mild anxiety and depression during the previous 30 days, while 12 (4.8%) and 13 (5.2%) had suffered from moderate and severe symptoms, respectively. Nineteen athletes (7.6%) reported that they had experienced suicidal ideation during the previous 2 weeks. Players with mental health problems experienced more events in competitions and daily life, including reduced subjective performance, missing opportunities to play during the last season, changes in health condition, and thinking about a career after retirement, compared with players without such problems. Mental health issues in Japan Rugby Top League players, as elite athletes, may be common, and research and practice development is expected in the near future.

## 1. Introduction

There is growing academic and practical interest in mental health support for elite athletes, including rugby players. Expert opinions and statements by several international sports committees have claimed that elite athletes also need mental health support to the same extent as the general population [1,2,3,4,5,6]. The pressing need to develop a mental health system for athletes, including early detection and intervention, has also been documented in expert opinions and statements.

These movements in mental health in sports are supported by the accumulated research findings of epidemiological surveys conducted in Australia, European countries, and the United States [7,8,9,10,11,12,13,14,15,16]. Previous papers showed that the prevalence of mental health symptoms in elite athletes is as common as in the general population of the same generation. According to a review paper by the International Olympic Committee (IOC), the prevalence of anxiety and depression in male elite athletes in team sports ranges from 5% to nearly 45% [5]. A meta-analysis reported a 33.6% prevalence of anxiety and depressive symptoms in current and former elite athletes [17]. In a previous study using samples similar to this current study, Nicholls et al. examined the prevalence and degree of anxiety and depression symptoms in UK professional rugby players [18], and they reported that 18.9% of athletes had mild and 13.7% had moderate/severe symptoms of anxiety. They also reported that the players showed mild (11.6%) and moderate/severe (2.6%) symptoms of depression [18].

The occurrence of suicidal ideation is considered to peak among adolescents and young adults, including elite athletes [19]. Suicidal ideation is strongly associated with mental health problems, and is also a high-risk factor for attempted and completed suicide [19]. Although there has been very little research into suicidal ideation in elite athletes, in a study of track and field athletes by Timpka et al., the authors demonstrated that about 15.6% of athletes (men 17.4%; women 14.2%) reported having experienced suicidal ideation [20]. In another survey of all deaths in US elite college student athletes, 7.3% were reported to be due to suicide [12]. Suicidality, including suicidal ideation, in elite athletes may be as common as in the general youth population. Risk factors associated with increased vulnerability to mental health symptoms include genetic, biological, and environmental factors that interact [21]. Early studies of competing athletes’ mental health risk factors adopted an exploratory approach, with several potential correlations broadly categorized as demographic characteristics and personal life events [5,22]. These studies suggested that the risk of mental health problems in athletes may be related to both sporting factors and non-sporting factors. Such accumulated research findings have been helpful in the development of athlete-specific mental health screening tools, for example, the Athlete Psychological Strain Questionnaire (APSQ) [23,24], as a mental health support measure for athletes.

Research and practice relating to mental health problems and the risk factors in elite athletes has increased rapidly, especially in Australia, European countries, and the United States [1,2,3,4,5,6]. Asian countries, including Japan, have published little on this topic. Therefore, little is known about the mental health status among elite athletes, including rugby players in Japan. The aim of this study is to examine the prevalence of anxiety and depression symptoms and suicidal ideation among a sample of Japan Rugby Top League players, and to also explore the risk factors for mental health problems in demographic characteristics and events experienced in competition and daily life. Reporting the mental health status of the players may contribute to developing a system for mental health promotion and support for elite athletes, including rugby players in Japan.

## 2. Materials and Methods

### 2.1. Study Design and Setting

We followed the STROBE guidelines for observational studies for the reporting of this cross-sectional study [25]. The Japan Rugby Players’ Association members launched the project for developing a mental health care system for Japan Rugby Top League players. As a part of this project, the current research design and this questionnaire were developed with reference to international studies, by a multidisciplinary team, including rugby players, psychiatrists, clinical psychologists, psychiatric social workers, public health nurses. The current study employed a web-based cross-sectional design. Questionnaires were distributed to all the athletes who enrolled in the Japan Rugby Players’ Association. The participants were invited to complete an anonymous online survey, which was submitted only by participants who voluntarily agreed. It took about 10 min to complete. The participants were informed via the cover page on the web about the process of the survey, including the purpose of the study, data collection procedures, and the consequences of participating or not participating in the study. The participants were reminded of any missing items prior to progressing to the next page, resulting in no missing outcome data. The participants were provided with individual one-time access using IP address filtering to a tablet or laptop computer to complete the survey. The cross-sectional data were collected in the off-season from December 2019 to January 2020. All investigators received the learning course on research ethics, this study was approved and facilitated by the Research Ethics Committee at the National Center of Neurology and Psychiatry (approval number: A2020-015).

### 2.2. Participants

We collected data provided by the Japan Rugby Players’ Association. The total of 600 male players registered were all aged 18 years and over. All the athletes belonged to the Japan Rugby Top League. Exclusion criteria were not applied. Out of this total, 251 participants gave consent (response rate: 41.8%). The response rate of this survey was not lower than other mental health surveys in Japan [26]. No patients were involved in this study. Members of the Japan Rugby Players’ Association were involved in the study design, implementation, reporting or dissemination plans of our research, together with the researchers.

### 2.3. Measures

The measures were selected by the Japan Rugby Football Players Association and mental health care experts, with reference to previous surveys [5,22] and priority was given to the use of validated scales. In this study, we used a web-survey to ask about mental health status and suicidal ideation, life events experienced, and demographic information. The survey also included items related to mental health knowledge, attitudes, and behavior. The components of the questionnaire relevant to this study are detailed below.

### 2.4. Anxiety and Depression Symptoms

The mental health status was assessed using the Japanese version of a globally widely accepted screening tool, the Kessler-6 (K6) [27,28]. This is a six-item self-report scale that evaluates the frequency of experiencing general psychological distress, such as nervousness, tiredness, hopelessness, and restlessness (e.g., “How often did you feel nervous?”, “How often did you feel hopeless?”). The scores are categorized to indicate the respondents’ mental health problems over the past 30 days. Responses to items are made on a 5-point scale. The K6 was developed and validated based on many epidemiological surveys, and is widely used as an indicator of severe mental disorders or mood and anxiety disorders. The score ranges from 0 to 24. High scores indicate more severe mental disorders. Previous studies have proposed that the level of psychological distress could be divided into the following four groups: no distress, mild, moderate, and severe distress. These are based on scores on the K6 scale of 0 to 4, 5 to 10, 10 to 12, and 13 to 24, respectively [29]. The K6 has been translated into Japanese. According to a study to establish the screening performance and optimal cut-off points for the Japanese version of K6, the optimal cut-off point was 4/5 on K6 with the sensitivity of 100% and moderate-to-high specificity [30]. The same study suggested the cut-off point of 12/13 for K6 can also be useful if screening targets severe mental illnesses [30]. Its validity in revealing mood and anxiety disorders in a community sample has been demonstrated and it has been confirmed to be a robust psychometric test in Japanese populations [28].

### 2.5. Suicidal Ideation

We used one item from the Baron Depression Screener for Athletes (BDSA) to reveal experience of suicidal ideation (i.e., I have thoughts of ending my life) [5,31]. The BDSA consists of a 10-item self-report questionnaire that addresses mood, sports-related anhedonia, weight loss, fatigue, self-image, substance abuse, suicidality, and other parameters over the previous 2 weeks, on a three-point Likert scale (range 0 to 20, a higher score representing more severe depressive symptoms). Utilizing the BDSA is recommended by the IOC’s consensus statement on mental health for elite athletes [5]. The scale has no cut-off scores that automatically suggest a diagnosis of major depressive disorder, according to Dr Baron, the developer of this scale [31]. We developed a Japanese version of BDSA (BDSA-J) and confirmed the one-factor structure of this scale to be the same as that of the original version of BDSA. In our previous research, BDSA-J showed significant and positive correlations with K6 (ρ = 0.489, *p* < 0.001) [32].

### 2.6. Life Events Experienced

Events experienced in competition and daily life in the past month were assessed with Yes (1) or No (0) for each of the following; fatigue, change in appetite, change in weight, sleep problems, alcohol-related troubles, move to another team, moving house, change in income, changes in family structure (additions: marriage, birth of a child, etc.) and losses: divorce, bereavement, etc.), childcare, socializing with friends, time for hobbies, changes in relationship with a spouse or partner, missing a game, reduced competitiveness, thinking about a career after retirement.

### 2.7. Statistical Methods

The data reported are derived from the mean score (standard deviation) of the scales and the prevalence of anxiety and depression symptoms and suicidal ideation. The percentage of players with specific demographic characteristics and experiences of certain life events was calculated according to anxiety and depression symptoms within the validated cut-off values of K6 (4/5 and 12/13, respectively) [30], and the presence or absence of suicidal ideation. As all variables were categorical and many cells expected its frequency less than 5, we conducted Fisher’s exact tests to consider all two-group differences. All analyses were conducted with Stata version 16 (StataCorp LLC, College Station, TX, USA). All tests were two-sided, and *p*-values were compared with the significance level of α = 0.05.

## 3. Results

Table 1 summarizes the players’ demographic characteristics and their experience of competition and daily life in the past month. A total of 51.8% of the players were 25–29 years old and 95.6% had a college degree. A total of 47.0% were married and 30.3% had children in their families. About half of them lived with their families and partners. A total of 21.9% of them reported that they had experience in the national team, and 35.9% did not play during the last season (Table 1).

The mean score for anxiety and depression symptoms across this sample was 4.4 (SD = 4.1). A total of 145 players (57.8%) had a normal score for anxiety and depression symptoms. 81 players (32.3%) reported mild symptoms. A total of 12 (4.8%) and 13 (5.2%) were suffering from moderate and severe symptoms, respectively. Nineteen players (7.6%) reported that they had experienced suicidal ideation.

Table 2 and Table 3 summarize the relationship of anxiety and depression symptoms and suicidal ideation to demographic characteristics and events experienced. Players who had severe anxiety and depression were more likely to be in reserve or not to have played during the last season than those who did not (Table 2). Regarding life events, players with anxiety and depression symptoms experienced significantly more fatigue, change in appetite, change in weight, sleep problems, alcohol-related troubles, change in income, and thinking about a career after retirement than those without such symptoms. Among these events, missing a game and reduced competitiveness were experienced more among athletes assessed as having severe anxiety and depression than those not. Players with suicidal ideation tended to have more change in appetite, change in weight, sleep problems, reduced competitiveness, and thinking about a career after retirement than those without suicidal ideation (Table 3).

## 4. Discussion

We have demonstrated the prevalence of anxiety and depression symptoms and suicidal ideation in Japan Rugby Top League players through a cross-sectional study. The current results demonstrate that the prevalence of mental health problems in the rugby players may be common. We also identified demographic characteristics and experiences that might pose a risk of potential mental health problems in competition and daily life.

The current results demonstrate that of the rugby players who participated, 3 to 4 in 10 experienced mild anxiety and depression symptoms. One in ten suffered from moderate or severe symptoms. According to previous studies [5], the mental health status of these players might be improved by the support of a mental health professional. The current results are comparable with a previous meta-analysis study in other countries, which reported a prevalence of anxiety and depressive symptoms of 33.6% in elite athletes [17]. It is also consistent with the 5 to 35% incidence of mental health problems in elite athletes reported in previous prospective studies, included in a review by Reardon et al. (2019) [5]. For Japanese elite athletes, including rugby players, experiencing mental health problems may be as common as elite athletes in other countries. Our findings suggest that mental health support for the players is also needed in Japan.

Regarding the prevalence of suicidal ideation, 7.6% of the rugby players in the Japan Rugby Top League reported such experience. The current results are lower than the 15.6% reported by Timpka et al. [20]. This inconsistency is due to the difference between our assessing the prevalence of suicidal ideation over the past two weeks, while Timpka et al. assessed the lifetime prevalence of suicidal ideation. A previous study that targeted young people (20–30s) in general in Japan reported that 7.3% had had suicidal ideation during the previous year [33]. Suicidal ideation of the rugby players is considered to be common as well as the general young people. According to the interpersonal theory of suicide [34], the players are considered to be high-risk to attempt suicide if they have suicidal ideation. This model demonstrated that suicidal ideation is driven by both “perceived burdensomeness” and “thwarted belongingness”. The third element, “acquired capability for suicide”, is conceived as an essential prerequisite for executing suicidal ideation and the desire to die and moving onto attempt suicide. Notably, the “acquired capability for suicide” is reciprocally reinforced by the habituation to the pain in the competitive athlete’s life and they are both involved in severe forms of suicidal attempts [35]. As other significant variables for the risk of suicidality, early life maltreatment and adverse childhood experience, including sexual abuse, had been reported in the previous review papers [36,37]. Suicidal prevention interventions should be needed to prevent athletes from suicidal ideation, which requires identifying athletes’ risk factors for the suicidal ideation.

Players with anxiety and depression symptoms and suicidal ideation had more experience of missing opportunities to play, life events, and changes in physical condition compared to players without such mental health problems. The risk factors we have found may be divided into those common to mental health problems and those specific to athletes [5,22]. For example, fatigue, appetite and weight changes, sleep problems, and alcohol-related troubles are risk factors for health conditions shared with the general public. Changing income, and thinking about a career after retirement are considered environmental risk factors. Missing a game and reduced competitiveness are athlete-specific factors. The experience of being reduced competitive and missing a match increases the burdensomeness toward the team and lead to a lack of belongingness. In addition, such experience can increase the burdensomeness toward their family. Concerns about decreasing competitiveness and retirement could be a crisis for athletes. Therefore, a longitudinal support system, including career development and support, should be needed. Several life events experienced might also be the result of behavior due to mental health problems. Drinking and excessive eating as impulsive stress coping, and interpersonal problems in social life due to aggressive behavior may particularly occur in groups with high masculinity including athletes. The current findings that situations and conditions that are evident in the daily lives of players are related to mental health problems may be useful for early detection and care by athletes themselves and their staff [38].

To the best of our knowledge, mental health research in elite athletes has been conducted in Australia, European countries, and the United States, with few reports from Asian countries. The current research is the first study to report on a sample of Japanese elite athletes, a different cultural sample from the previous findings. We also recognize several limitations that should be taken into consideration. First, we assessed all-male rugby players, because this was feasible based on the data available in this survey. Although the response rate of this survey was about 40%, which is no lower than other mental health surveys [26], the rate may not be sufficient to accurately estimate the overall trend among elite athletes in Japan. To better generalize the findings of this study, future research should assess the mental health status in other sports and among female elite athletes. In addition, given that there may be significant cross-cultural differences in the meaning and symptoms of mental health symptoms and illnesses [39], cross-cultural or national studies are required. Second, the data we analyzed is cross-sectional and was collected at one point in time. Ideally, a long-term, prospective study would address these challenges. In addition, the timing of conducting a survey might affect mental health status, whether during the competitive season or the off-season. Third, the K6, which we used in this study, is an assessment scale for general psychological symptoms, especially internalizing symptoms. In populations that tend to be predominantly male, such as rugby players, externalizing symptoms (e.g., substance abuse, risk-taking, or poor impulse control) are more often observed than internalizing symptoms [40,41]. Therefore, other scales including externalizing symptoms might lead to different results. Recently, Rice et al. developed the new athlete-specific mental health screening tool, the Athlete Psychological Strain Questionnaire (APSQ) [23,24], including the items of externalizing symptoms and subjective performance. APSQ has been adopted in IOC mental health screening tools [42]. Fourth, the variables related to risk factors of mental health problems in the athlete examined in this study might not be sufficient. In addition, in the current findings, since the data were obtained by a cross-sectional design for describing the mental health status in the population, we would not establish cause-and-effect relationships.

## 5. Conclusions

While globally rising clinical and research interests are driving the move to promote and support the mental health of elite athletes, only a small number of fundamental epidemiological studies, as the first step for the innovation, have investigated mental health issues. We have reported that mental health problems, including anxiety and depression symptoms and/or suicidal ideation, may be common to Japan Rugby Top League players and elite athletes. Mental health problems may be associated with the experience of events in competition and daily life. This information should be shared among players, their coaches, health professionals and others working in sports society for early detection and support. Our efforts may contribute to developing mental health services for elite athletes in the near future.

## Figures and Tables

**Table 1:** Demographic characteristics of the study participants and their experience of life events.

Variable	% (*n*)
Age at survey	
~19	0.40 (1)
20~24	19.92 (50)
25~29	51.79 (130)
30~34	24.70 (62)
35~	3.19 (8)
Educational attainment	
High school	2.79 (7)
Four-year college or university	95.62 (240)
Postgraduate college (or more)	1.59 (4)
Marital status	
Married	47.01 (118)
Never married	51.00 (128)
Divorced or widowed	1.99 (5)
Child living in household	30.28 (76)
Residential Status	
Living alone	19.52 (49)
With family or partner	50.20 (126)
Dormitory	30.28 (76)
Experience of national team	21.91 (55)
Player status during last season	
As a starting member	32.27 (81)
As a reserve member	31.89 (80)
No play	35.86 (90)
Life events experienced	
Fatigue	90.44 (227)
Change in appetite	52.59 (132)
Change in weight	59.36 (149)
Sleep problems	60.96 (153)
Alcohol related troubles	23.11 (58)
Move to another team	15.14 (38)
Moving house	18.73 (47)
Change in income	32.27 (81)
Changes in family structure (Additions: Marriage, birth of a child, etc.)	23.11 (58)
Changes in family structure (Losses: Divorce, bereavement, etc.)	12.35 (31)
Childcare	28.69 (72)
Socializing with friends	74.50 (187)
Time for hobbies	76.89 (193)
Changes in relationship with spouse or partner	54.98 (138)
Missing a game (did not play in the game)	69.32 (174)
Reduced competitiveness	59.36 (149)
Thinking about a career after retirement	70.12 (176)

**Table 2:** Relationship of anxiety and depression symptoms and suicidal ideation to demographic characteristics.

Factor	K6 Score						Suicidal Ideation		
	5<	≥5	*p* Value	13<	≥13	*p* Value	Without	With	*p* Value
	% (*n*)	% (*n*)		% (*n*)	% (*n*)		% (*n*)	% (*n*)	
**Age at survey**									
~19	0.0 (0)	0.9 (1)	0.63	0.0 (0)	7.7 (1)	0.08	0.4 (1)	0.0 (0)	0.51
20~24	22.1 (32)	17.0 (18)		19.3 (46)	30.8 (4)		19.0 (44)	31.6 (6)	
25~29	51.7 (75)	51.9 (55)		52.1 (124)	46.2 (6)		51.7 (120)	52.6 (10)	
30~34	22.8 (33)	27.4 (29)		25.2 (60)	15.4 (2)		25.4 (59)	15.8 (3)	
35~	3.5 (5)	2.83 (3)		3.4 (8)	0.0 (0)		3.5 (8)	0.0 (0)	
**Educational attainment**									
High school	4.8 (7)	0 (0)	0.03 ^a^	2.9 (7)	0.0 (0)	1.00	3.0 (7)	0.0 (0)	0.62
Four-year college or university	93.1 (135)	99.1 (105)		95.4 (227)	100.0 (13)		95.3 (221)	100.0 (19)	
Postgraduate college (or more)	2.1 (3)	0.94 (1)		1.7 (4)	0.0 (0)		1.7 (4)	0.0 (0)	
**Marital status**									
Never married	49.7 (72)	43.4 (46)	0.46	46.2 (110)	61.5 (8)	0.54	46.1 (107)	57.9 (11)	0.65
Married	49.0 (71)	53.8 (57)		51.7 (123)	38.5 (5)		51.7 (120)	42.1 (8)	
Divorced or widowed	1.4 (2)	2.8 (3)		2.1 (5)	0.0 (0)		2.2 (5)	0.0 (0)	
Child living in household	29.7 (43)	31.1 (33)	0.90	30.7 (73)	23.1 (3)	0.76	30.2 (70)	31.6 (6)	1.00
**Residential Status**									
Living alone	20.7 (30)	17.9 (19)	0.47	20.2 (48)	7.7 (1)	0.61	19.0 (44)	26.3 (5)	0.74
With family or partner	46.9 (68)	54.7 (58)		50.0 (119)	53.9 (7)		50.4 (117)	47.4 (9)	
Dormitory	32.4 (47)	27.4 (29)		29.8 (71)	38.5 (5)		30.6 (71)	26.3 (5)	
Experience of national team	19.3 (28)	25.5 (27)	0.28	22.3 (185)	15.4 (2)	0.43	21.1 (49)	31.6 (6)	0.38
**Player status during last season**									
As a starting member	31.7 (46)	33.0 (35)	0.99	34.0 (81)	0.0 (0)	**0.01 ^a^**	33.2 (77)	21.1 (4)	0.43
As a reserve member	32.4 (47)	31.1 (33)		31.5 (75)	38.5 (5)		31.9 (74)	31.6 (6)	
No play	35.9 (52)	35.9 (38)		34.5 (82)	61.5 (8)		34.9 (81)	47.4 (9)	

**Bold**: significant at *p* < 0.016; a: Bonferroni correction was applied for significance (α = 0.05/3 = 0.016).

**Table 3:** Relationship of anxiety and depression symptoms and suicidal ideation to life events experience.

Factor	K6 Score						Suicidal Ideation		
	5<	≥5	*p* Value	13<	≥13	*p* Value	Without	With	*p* Value
	% (*n*)	% (*n*)		% (*n*)	% (*n*)		% (*n*)	% (*n*)	
**Life events experienced**									
Fatigue	84.8 (123)	98.1 (104)	**0.00**	90.3 (215)	92.3 (12)	1.00	90.1 (209)	94.7 (18)	1.00
Change in appetite	46.2 (67)	61.3 (65)	**0.02**	52.1 (124)	61.5 (8)	0.58	50.4 (117)	79.0 (15)	**0.02**
Change in weight	53.1 (77)	67.9 (72)	**0.02**	58.8 (140)	69.2 (9)	0.57	57.3 (133)	84.2 (16)	**0.03**
Sleep problems	49.0 (71)	77.4 (82)	**0.00**	60.5 (144)	69.2 (9)	0.77	58.6 (136)	89.5 (17)	**0.01**
Alcohol related troubles	17.9 (26)	30.2 (32)	**0.03**	22.3 (53)	38.5 (5)	0.19	21.6 (50)	42.1 (8)	0.05
Move to another team	14.5 (21)	16.0 (17)	0.73	14.7 (35)	23.1 (3)	0.42	15.1 (35)	15.8 (3)	1.00
Moving house	17.2 (25)	20.8 (22)	0.52	17.7 (42)	38.5 (5)	0.07	18.5 (43)	21.1 (4)	0.76
Change in income	26.9 (39)	39.6 (42)	**0.04**	31.5 (75)	46.2 (6)	0.36	31.5 (73)	42.1 (8)	0.44
Changes in family structure (Additions: Marriage, birth of a child, etc.)	20.7 (30)	26.4 (28)	0.29	22.3 (53)	38.5 (5)	0.19	22.8 (53)	26.3 (5)	0.78
Changes in family structure (Losses: Divorce, bereavement, etc.)	11.0 (16)	14.2 (15)	0.56	11.3 (27)	30.8 (4)	0.06	12.1 (28)	15.8 (3)	0.71
Childcare	26.2 (38)	32.1 (34)	0.33	27.7 (66)	46.2 (6)	0.21	28.5 (66)	31.6 (6)	0.79
Socializing with friends	70.3 (102)	80.2 (85)	0.08	74.4 (177)	76.9 (10)	1.00	73.3 (170)	89.5 (17)	0.17
Time for hobbies	73.8 (107)	81.1 (86)	0.23	76.9 (183)	76.9 (10)	1.00	76.3 (177)	84.2 (16)	0.58
Changes in relationship with spouse or partner	51.7 (75)	59.4 (63)	0.25	55.0 (131)	53.9 (7)	1.00	53.9 (125)	68.4 (13)	0.24
Missing a game	62.1 (90)	79.3 (84)	**0.00**	67.7 (161)	100.0 (13)	**0.01**	69.0 (160)	73.7 (14)	0.80
Reduced competitiveness	49.0 (71)	73.6 (78)	**0.00**	57.6 (137)	92.3 (12)	**0.02**	56.5 (131)	94.7 (18)	**0.00**
Thinking about a career after retirement	64.1 (93)	78.3 (83)	**0.02**	68.9 (164)	92.3 (12)	0.12	68.1 (158)	94.7 (18)	**0.02**

**Bold**: significant at *p* < 0.05.

## Data Availability

Not all the data are freely accessible because no informed consent was given by the participants for open data sharing, but we can provide the data used in this study to researchers who want to use them, following approval by the ethics committee of NCNP.
